# Declining laser peripheral iridotomy for angle closure alongside rising cataract surgeries: A nationwide cohort study in South Korea

**DOI:** 10.1371/journal.pone.0343427

**Published:** 2026-02-20

**Authors:** Woojin Kim, Chan Mi Park, Seokjin Kong, Dong Hyun Kim, Youngsub Eom, Jong Suk Song

**Affiliations:** 1 Department of Ophthalmology, Korea University Guro Hospital, Seoul, Republic of Korea; 2 Biomedical Research center, Korea University Guro Hospital, Seoul, Republic of Korea; 3 Department of Biomedical Informatics, Korea University College of Medicine, Seoul, Republic of Korea; 4 Department of Ophthalmology, Korea University Anam Hospital, Seoul, Republic of Korea; 5 Department of Ophthalmology, Korea University Ansan Hospital, Gyeonggi-do, Republic of Korea; Hangil Eye Hospital / Catholic Kwandong University College of Medicine, KOREA, REPUBLIC OF

## Abstract

**Purpose:**

To examine recent nationwide trends in cataract surgeries and laser peripheral iridotomy (LPI) in Korea, and to assess their temporal association and patient-level characteristics influencing procedure patterns.

**Methods:**

This retrospective cohort study used data from the Korean National Health Information Database. Individuals aged 65 years and older who underwent cataract surgery or LPI between 2016 and 2021 were analyzed. Primary outcomes included the annual volumes of cataract surgeries and LPIs, their temporal association, patient demographics, the proportion of LPI patients subsequently undergoing cataract surgery, and the interval to surgery.

**Results:**

Cataract surgeries increased annually until 2019, declined in 2020, and rose again in 2021, whereas LPI procedures steadily decreased after 2017. A negative correlation was observed between annual cataract surgeries and LPIs (*r* = −0.657, *P* = 0.156), although not statistically significant. Among patients aged 65–74 years, the correlation was significant (*r* = −0.943, *P* = 0.005). Among those who underwent LPI, 92.1% subsequently received cataract surgery, with a median interval of 121 days between procedures.

**Conclusion:**

This nationwide study demonstrated a temporal association and demographic characteristics underlying the recent increase in cataract surgeries and the concurrent decline in LPI procedures in Korea. Notably, most patients who underwent LPI subsequently received cataract surgery within a relatively short interval, suggesting a possible clinical shift toward earlier lens extraction in the management of angle closure.

## Introduction

Cataract and glaucoma are the two leading causes of global blindness, accounting for 15.2 million and 3.6 million cases, respectively, among individuals aged 50 years and older in 2020 [1,2]. While cataract-induced visual impairment can be effectively treated with surgery [2,3], glaucomatous optic neuropathy can lead to irreversible blindness [4]. Glaucoma has several spectrums, and among them, primary angle-closure glaucoma (PACG) is less common than primary open-angle glaucoma, yet it is more prevalent in Asia and causes more severe visual impairment [5,6]. Primary angle-closure (PAC) is an early stage of PACG, characterized by the absence of glaucomatous optic neuropathy and no visual impairment [5,7].

The traditional treatment for PAC or PACG includes laser peripheral iridotomy (LPI) and medical care using eye drops to reduce intraocular pressure (IOP) [7]. LPI opens the drainage pathways, relieves pupillary block, and transiently widens the anterior chamber angle; however, its effectiveness declines over time [5,8]. Therefore, in cases of PAC or PACG where a cataract is present, lens extraction through cataract surgery is often considered before performing LPI [8,9]. Furthermore, even in the absence of a cataract, clear lens extraction itself is reported to be effective in treating PAC or PACG [5,7].

In South Korea, cataract surgeries have increased in recent years, partly due to expanded use of premium intraocular lenses (IOLs) supported by private health insurance [10]. This shift toward earlier lens-based intervention—particularly among younger elderly adults—may have contributed to a decreased reliance on LPI procedures. We investigated nationwide trends in cataract surgeries and LPI over the past six years, analyzing their correlation and evaluating associated demographic factors and clinical outcomes. This is the first nationwide, large-scale study of these trends during the COVID-19 pandemic era. It also provides novel data on the proportion and timing of cataract surgeries following LPI.

## Methods

This nationwide, population-based, retrospective cohort study used information from the National Health Information Database (NHID) created by the Korean National Health Insurance Service (KNHIS). The study period (2016–2021) was selected to provide an up-to-date analysis of recent real-world practice patterns based on the most current nationally available data. The dataset was formally requested from the NHID on November 14, 2022, and analyses were conducted between June 2023 and March 2024. Only de-identified data were provided, and the authors had no access to personally identifiable information at any stage. This study was approved by the Institutional Review Board (IRB) of the affiliated institution (IRB no. 2022GR0364) and adhered to the tenets of the Declaration of Helsinki. Informed consent was waived due to the retrospective design.

In South Korea, the KNHIS is a single medical care system that covers nearly all Koreans; 97% are NHIS beneficiaries who pay an insurance premium, and 3% are recipients of the government’s Medical Aid program [10]. NHID information is created when medical institutions submit claims to the insurer for medical services provided to patients. The NHID contains a vast amount of data, including patients’ demographic data and health claims data. Diagnostic codes are defined by the Korean Standard Classification of Diseases, Eighth Revision (KCD-8), which aligns with the International Classification of Diseases, Tenth Revision. Therefore, studies using NHID information have the capability to analyze nearly all data associated with medical care conducted in Korea.

### Study population

**Fig 1:**
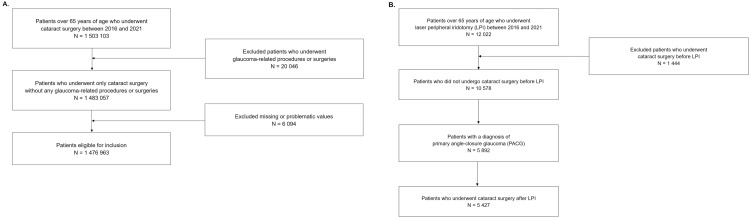
Flowchart of study population selection. **(A)** Patients who underwent cataract surgery. **(B)** Patients who underwent laser peripheral iridotomy (LPI).

The study population consisted of all patients aged 65 years or older who underwent cataract surgery (n = 1,503,103) or LPI (n = 12,022) in hospitals in South Korea between January 1, 2016, and December 31, 2021 (Fig 1). The patient cohort was selected to be over 65 years of age because PACG predominantly affects older individuals [11]. Cataract surgery was identified using the medical practice code S5117 (Intraocular Lens Implantation—Primary [Simultaneous with Cataract Surgery]), and LPI was identified using the medical practice code S5041 (Surgery for Glaucoma-Iridectomy) [11]. PACG was analyzed using H40.2 of the KCD-8 codes. In the cataract surgery group, patients who underwent glaucoma-related procedures or surgeries before cataract surgery (n = 20,046) were excluded, as were cases with missing values or more than three cataract surgery codes (n = 6,094) (Fig 1A). In the LPI group, patients who underwent cataract surgery prior to LPI (n = 1,444) were excluded. Among the remaining patients (n = 10,578), those who also had a PACG diagnosis (n = 5,892) were analyzed, including patients who underwent cataract surgery after LPI (n = 5,427) (Fig 1B). Because the NHID does not provide laterality information, all analyses were performed at the person-level, and eye-specific relationships could not be determined.

### Independent variables

The annual number of patients who underwent cataract surgery or LPI for PACG was analyzed, along with the rate of these procedures per 100,000 persons aged 65 years or older. Additionally, the correlation between yearly trends in cataract surgery and LPI was assessed. In both groups, factors such as age, sex, residence, and the type of hospital where the surgery or procedure was performed were evaluated. Ocular comorbidities were analyzed using KCD-8 codes for diabetic retinopathy (H36.0*) and age-related macular degeneration (AMD) (H35.3). Systemic comorbidity was assessed using the Charlson Comorbidity Index (CCI), a validated weighted score summarizing a patient’s overall systemic disease burden based on predefined medical conditions [12].

We also analyzed the proportion of patients who underwent cataract surgery after LPI, as well as the interval between LPI and the first cataract surgery. The time from LPI to the first cataract surgery was categorized as within 6 months (up to 180 days), 6 months to one year (181–365 days), one to two years (366–730 days), and two years or more (731 days or more). The ratios and characteristics for each time category were then analyzed.

### Statistical analysis

The data were expressed as mean ± standard deviation values for continuous variables and n (%) for categorical variables. The primary outcome was the number of patients who underwent cataract surgery and LPI by year. For the calculation of the rate per 100,000 population, we used the annual population of adults aged 65 years and older obtained from Statistics Korea. Given the limited number of annual time points, we used Spearman’s rank correlation analysis as an exploratory, non-parametric method to assess the monotonic association between the annual volumes of cataract surgeries and LPI procedures, without assuming linearity. Descriptive statistics were presented separately for the cataract surgery and LPI groups, as the two groups represent distinct clinical populations with differing indications for intervention. Accordingly, no direct statistical comparisons between the two groups were performed. In cataract surgery or LPI patients, the Jonckheere–Terpstra test was used to evaluate monotonic distributional trends of Age and CCI across ordered calendar years, while the Cochran-Mantel-Haenszel test was employed to assess the associations among age groups, types of hospitals, and CCI groups by year. Additionally, the Cochran-Armitage trend test was used to evaluate linear trends in sex, region of residence, and ocular comorbidity over the years. For patients who underwent cataract surgery after LPI, categorized by ordered duration groups, mean differences in age and CCI across the four ordered interval groups were evaluated using one-way ANOVA. Other categorical characteristics were analyzed using the chi-squared test. All statistical analyses were performed using Statistical Analysis Software, version 9.4 (SAS Institute, Cary, NC, USA). P < 0.05 was considered statistically significant.

## Results

**Fig 2:**
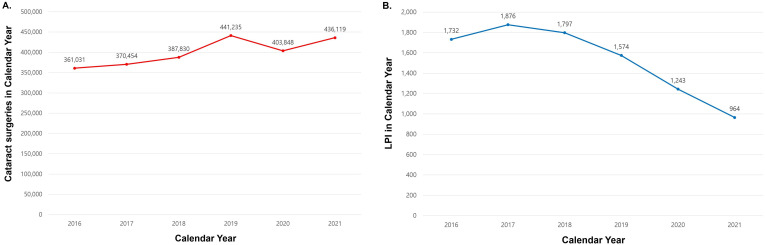
Annual number of cataract surgeries and LPIs. **(A)** Cataract surgeries. **(B)** LPIs.

In Korea, from January 2016 to December 2021, the total number of cataract surgeries performed on individuals aged 65 and older was 2,400,517 (1,476,963 patients), and the total number of LPI procedures was 9,186 (5,892 patients) (Table 1 and Table 2). The number of cataract surgeries per year increased from 361,031 in 2016 to a peak of 441,235 in 2019, declined to 403,848 in 2020, and rebounded to 436,119 in 2021 (Fig 2A). Similarly, the number of cataract surgeries per 100,000 individuals aged 65 years and older rose from 2,621 in 2017–2,869 in 2019, declined to 2,477 in 2020, and then increased again to 2,544 in 2021 (detailed annual rates: [2016: 2,672], [2017: 2,621], [2018: 2,633], [2019: 2,869], [2020: 2,477], [2021: 2,544]). The number of LPI procedures per year ranged from 1,732 in 2016–964 in 2021, indicating a steady decline after 2017 (Fig 2B). Likewise, the number of LPI procedures per 100,000 individuals aged 65 years and older decreased consistently from 13.27 in 2017 to 5.62 in 2021 (annual rates: [2016: 12.82], [2017: 13.27], [2018: 12.2], [2019: 10.24], [2020: 7.62], [2021: 5.62]). The data suggested a trend of a negative correlation between the annual numbers of cataract surgeries and LPIs, but this correlation did not reach statistical significance (Spearman’s rank correlation coefficient [*r*] = −0.657, *P* = 0.156). When classified by age, a statistically significant negative correlation was observed in those aged 65–74 (*r* = −0.943, *P* = 0.005), while a non-significant positive correlation was found in those aged 75 or older (*r* = 0.429, *P* = 0.397). By sex, negative correlations were observed in both males (*r* = −0.543, *P* = 0.266) and females (*r* = −0.771, *P* = 0.072), with a stronger trend in females, although neither reached statistical significance.

**Table 1:** Characteristics of cataract surgery between 2016 and 2021.

		Calendar year						*P* value for trend
**Characteristic**	**All**	**2016**	**2017**	**2018**	**2019**	**2020**	**2021**	
**No. of eyes**	2,400,517	361,031	370,454	387,830	441,235	403,848	436,119	
**No. of patients**	1,476,963	246,435	238,779	241,135	265,326	235,397	249,891	
**Age (yrs)**								
**Median (IQR)**	74 (69-78)	74 (70-78)	74 (70-78)	74 (70-78)	74 (69-78)	73 (69-78)	73 (69-78)	<0.0001*
**Mean (SD)**	74.18 (5.99)	74.45 (5.81)	74.44 (5.93)	74.38 (5.96)	74.31 (6.02)	73.76 (6.03)	73.75 (6.12)	
**65-74 [n (%)]**	801,764 (54.28)	129,907 (52.71)	122,696 (51.38)	125,782 (52.16)	140,928 (53.12)	136,420 (57.95)	146,031 (58.44)	<0.0001*
**75-84**	597,542 (40.46)	103,934 (42.18)	103,271 (43.25)	102,455 (42.49)	109,846 (41.40)	87,337 (37.10)	90,699 (36.30)	
**85+**	77,657 (5.26)	12,594 (5.11)	12,812 (5.37)	12,898 (5.35)	14,552 (5.48)	11,640 (4.94)	13,161 (5.27)	
**Sex**								<0.0001*
**Male**	608,771 (41.22)	97,578 (39.60)	96,847 (40.56)	99,513 (41.27)	109,753 (41.37)	99,209 (42.15)	105,871 (42.37)	
**Female**	868,192 (58.78)	148,857 (60.40)	141,932 (59.44)	141,622 (58.73)	155,573 (58.63)	136,188 (57.85)	144,020 (57.63)	
**Region of residence**								<0.0001*
**Metropolitan**	646,075 (43.74)	103,540 (42.02)	102,596 (42.97)	105,563 (43.78)	116,895 (44.06)	105,642 (44.88)	111,839 (44.76)	
**Rural**	830,888 (56.26)	142,895 (57.98)	136,183 (57.03)	135,572 (56.22)	148,431 (55.94)	129,755 (55.12)	138,052 (55.24)	
**Type of hospital**								<0.0001*
**Tertiary hospital**	167,850 (11.36)	21,877 (8.88)	23,673 (9.91)	27,266 (11.31)	36,010 (13.57)	28,267 (12.01)	30,757 (12.31)	
**General hospital**	71,463 (4.84)	15,882 (6.44)	12,677 (5.31)	10,973 (4.55)	10,280 (3.87)	10,230 (4.35)	11,421 (4.57)	
**Hospital**	123,242 (8.34)	19,333 (7.85)	20,719 (8.68)	22,415 (9.30)	20,997 (7.91)	19,524 (8.29)	20,254 (8.11)	
**Clinic and others**	1,114,408 (75.45)	189,343 (76.83)	181,710 (76.10)	180,481 (74.85)	198,039 (74.64)	177,376 (75.35)	187,459 (75.02)	
**Ocular comorbidity**								
**Diabetic retinopathy**	107,159 (7.26)	18,206 (7.39)	17,314 (7.25)	17,804 (7.38)	19,160 (7.22)	16,994 (7.22)	17,681 (7.08)	<0.0001*
**AMD**	162,601 (11.01)	21,407 (8.69)	22,515 (9.43)	25,887 (10.74)	31,850 (12.00)	30,814 (13.09)	30,128 (12.06)	<0.0001*
**CCI**								
**Median (IQR)**	0 (0-1)	0 (0-1)	0 (0-1)	0 (0-1)	0 (0-1)	0 (0-1)	0 (0-1)	<0.0001*
**Mean (SD)**	0.83 (1.12)	0.84 (1.11)	0.84 (1.12)	0.84 (1.12)	0.84 (1.13)	0.82 (1.12)	0.78 (1.10)	
**0-2**	1,355,323 (91.76)	225,550 (91.53)	218,776 (91.62)	220,762 (91.55)	242,899 (91.55)	216,325 (91.90)	231,011 (92.44)	<0.0001*
**3-6**	118,873 (8.05)	20,498 (8.32)	19,568 (8.20)	19,933 (8.27)	21,913 (8.26)	18,584 (7.89)	18,377 (7.35)	
**≥ 7**	2,767 (0.19)	387 (0.16)	435 (0.18)	440 (0.18)	514 (0.19)	488 (0.21)	503 (0.20)	

*AMD* age-related macular degeneration, *CCI* Charlson comorbidity index, *IQR* interquartile range, *SD* standard deviation.

Data are presented as no. of patients (%), unless otherwise specified.

*Statistically significant

**Table 2:** Characteristics of laser peripheral iridotomy between 2016 and 2021.

		Calendar year						*P* value for trend
**Characteristic**	**All**	**2016**	**2017**	**2018**	**2019**	**2020**	**2021**	
**No. of eyes**	9,186	1,732	1,876	1,797	1,574	1,243	964	
**No. of patients**	5,892	1,266	1,293	1,087	951	744	551	
**Age (yrs)**								
**Median (IQR)**	73 (69-77)	73 (69-77)	72 (69-76)	73 (69-77)	72 (68-77)	73 (69-78)	73 (69-78)	0.065
**Mean (SD)**	73.30 (5.75)	73.00 (5.56)	72.89 (5.44)	73.30 (5.65)	73.37 (5.96)	73.70 (6.13)	73.70 (6.09)	
**65-74 [n (%)]**	3,580 (60.76)	786 (62.09)	818 (63.26)	646 (59.43)	566 (59.52)	439 (59.01)	325 (58.98)	0.009*
**75-84**	2,066 (35.06)	431 (34.04)	438 (33.87)	403 (37.07)	341 (35.86)	258 (34.68)	195 (35.39)	
**85+**	246 (4.18)	49 (3.87)	37 (2.86)	38 (3.50)	44 (4.63)	47 (6.32)	31 (5.63)	
**Sex**								0.565
**Male**	1,579 (26.80)	325 (25.67)	350 (27.07)	295 (27.14)	261 (27.44)	202 (27.15)	146 (26.50)	
**Female**	4,313 (73.20)	941 (74.33)	943 (72.93)	792 (72.86)	690 (72.56)	542 (72.85)	405 (73.50)	
**Region of residence**								0.013*
**Metropolitan**	2,916 (49.49)	623 (49.21)	709 (54.83)	514 (47.29)	454 (47.74)	369 (49.60)	247 (44.83)	
**Rural**	2,976 (50.51)	643 (50.79)	584 (45.17)	573 (52.71)	497 (52.26)	375 (50.40)	304 (55.17)	
**Type of hospital**								<0.0001*
**Tertiary hospital**	1,573 (26.70)	227 (17.93)	316 (24.44)	292 (26.86)	329 (34.60)	217 (29.17)	192 (34.85)	
**General hospital**	630 (10.69)	205 (16.19)	115 (8.89)	85 (7.82)	74 (7.78)	91 (12.23)	60 (10.89)	
**Hospital**	838 (14.22)	147 (11.61)	173 (13.38)	189 (17.39)	115 (12.09)	123 (16.53)	91 (16.52)	
**Clinic and others**	2,851 (48.39)	687 (54.27)	689 (53.29)	521 (47.93)	433 (45.53)	313 (42.07)	208 (37.75)	
**Ocular comorbidity**								
**Diabetic retinopathy**	247 (4.19)	63 (4.98)	57 (4.41)	38 (3.50)	45 (4.73)	31 (4.17)	13 (2.36)	0.051
**AMD**	398 (6.75)	73 (5.77)	88 (6.81)	82 (7.54)	72 (7.57)	48 (6.45)	35 (6.35)	0.499
**CCI**								
**Median (IQR)**	0 (0-1)	0 (0-1)	0 (0-1)	0 (0-1)	0 (0-1)	0 (0-1)	0 (0-1)	0.001*
**Mean (SD)**	0.71 (1.02)	0.76 (1.04)	0.75 (1.05)	0.69 (0.99)	0.65 (0.99)	0.70 (1.01)	0.64 (0.97)	
**0-2**	5,530 (93.86)	1,182 (93.36)	1,209 (93.50)	1,028 (94.57)	895 (94.11)	696 (93.55)	520 (94.37)	0.951
**3-6**	354 (6.01)	82 (6.48)	82 (6.34)	58 (5.34)	55 (5.78)	46 (6.18)	31 (5.63)	
**≥ 7**	8 (0.14)	2 (0.16)	2 (0.15)	1 (0.09)	1 (0.11)	2 (0.27)	0 (0)	

*AMD* age-related macular degeneration, *CCI* Charlson comorbidity index, *IQR* interquartile range, *SD* standard deviation.

Data are presented as no. of patients (%), unless otherwise specified.

*Statistically significant

The baseline characteristics of cataract surgery from 2016 to 2021 are shown in Table 1, while those of LPI are presented in Table 2. As the cataract surgery and LPI groups represent distinct clinical populations with fundamentally different indications for intervention, no direct statistical comparisons between these groups were performed. The mean age of patients who underwent cataract surgery was 74.18 ± 5.99 years, and the mean age of those who received LPI was similar at 73.30 ± 5.75 years. Among the cataract surgery patients, there were 608,771 male patients (41.2%) and 868,192 female patients (58.8%). In the LPI group, there were 1,579 male patients (26.8%) and 4,313 female patients (73.2%), indicating a notably higher proportion of women receiving LPI treatment. Among patients who underwent cataract surgery, 646,075 (43.7%) resided in metropolitan cities, and for those who underwent LPI, 2,916 (49.5%) resided in metropolitan cities. Among cataract surgery patients, 1,114,408 (75.5%) received the procedure in clinics, 167,850 (11.4%) in tertiary general hospitals, 123,242 (8.3%) in hospitals, and 71,463 (4.8%) in general hospitals. Among LPI patients, 2,851 (48.4%) received the procedure in clinics, 1,573 (26.7%) in tertiary general hospitals, 838 (14.2%) in hospitals, and 630 (10.7%) in general hospitals. Among patients who underwent cataract surgery, 107,159 (7.3%) had diabetic retinopathy, and 162,601 (11.0%) had AMD. Among patients who received LPI, 247 (4.2%) had diabetic retinopathy, and 398 (6.8%) had AMD. Regarding systemic comorbidities, the proportion of patients with CCI scores of 0–2 was the highest in both groups (1,355,323 [91.8%] and 5,530 [93.9%]).

Among the patients who received LPI with a diagnosis of PACG (n = 5,892), 5,427 (92.1%) underwent cataract surgery after the procedure (Fig 1B). Table 3 displays the characteristics of these patients. Age distributions were comparable between patients who underwent cataract surgery after LPI and those who underwent LPI alone (mean 74.20 ± 5.64 vs. 73.30 ± 5.75 years). The mean interval between LPI and the first cataract surgery was 339.6 ± 436.9 days, with a first quartile of 29 days, a median of 121 days, and a third quartile of 521 days. The interval to the first cataract surgery after LPI was within 180 days for 3,028 patients, between 181 and 365 days for 660 patients, between 366 and 730 days for 784 patients, and over 731 days for 955 patients. As with the overall group of patients who underwent LPI, those who underwent cataract surgery after LPI also had a higher proportion of female patients (3,990 [73.5%]), a relatively lower proportion of procedures performed in clinics (2,643 [48.7%]), and a higher proportion performed in tertiary hospitals (1,481 [27.3%]). Patients who underwent cataract surgery after LPI had a higher rate of AMD (707 [13.0%]) than patients who underwent LPI alone (398 [6.8%]). Patients who underwent cataract surgery after LPI also had the highest rate of CCI scores of 0–2 (5,042 [92.9%]). P values were calculated to assess whether patient characteristics varied across the four interval groups. While some comparisons were statistically significant, the differences were small and not considered clinically meaningful. Among the patients who underwent cataract surgery after LPI, 195 patients (3.6%) received a trabeculectomy, and 59 patients (1.1%) received an Ahmed valve implantation.

**Table 3:** Cataract surgery proportion and duration after laser peripheral iridotomy.

		Interval between LPI and the first cataract surgery				*P* value
**Characteristics** **at the time of cataract surgery**	**All**	**0-180 days**	**181-365 days**	**366-730 days**	**≥731 days**	
**No. of patients**	5,427	3,028	660	784	955	
**Age (yrs)**						
**Median (IQR)**	74 (70-78)	73 (69-78)	73 (69-77)	74 (70-78)	74 (71-78)	
**Mean (SD)**	74.20 (5.64)	74.04 (5.95)	73.71 (5.44)	74.24 (5.20)	75.03 (5.04)	<0.0001*
**65-74 [n (%)]**	3,003 (55.33)	1,699 (56.11)	380 (57.58)	431 (54.97)	493 (51.62)	0.002*
**75-84**	2,169 (39.97)	1,162 (38.38)	255 (38.64)	328 (41.84)	424 (44.40)	
**85+**	255 (4.70)	167 (5.52)	25 (3.79)	25 (3.19)	38 (3.98)	
**Sex**						0.188
**Male**	1,437 (26.48)	782 (25.83)	192 (29.09)	221 (28.19)	242 (25.34)	
**Female**	3,990 (73.52)	2,246 (74.17)	468 (70.91)	563 (71.81)	713 (74.66)	
**Region of residence**						0.111
**Metropolitan**	2,695 (49.66)	1,481 (48.91)	313 (47.42)	397 (50.64)	504 (52.77)	
**Rural**	2,732 (50.34)	1,547 (51.09)	347 (52.58)	387 (49.36)	451 (47.23)	
**Type of hospital**						0.001*
**Tertiary hospital**	1,481 (27.29)	821 (27.11)	190 (28.79)	227 (28.95)	243 (25.45)	
**General hospital**	531 (9.78)	325 (10.73)	61 (9.24)	66 (8.42)	79 (8.27)	
**Hospital**	772 (14.23)	471 (15.55)	92 (13.94)	99 (12.63)	110 (11.52)	
**Clinic and others**	2,643 (48.70)	1,411 (46.60)	317 (48.03)	392 (50.00)	523 (54.76)	
**Ocular comorbidity**						
**Diabetic retinopathy**	350 (6.45)	169 (5.58)	54 (8.18)	66 (8.42)	61 (6.39)	0.007*
**AMD**	707 (13.03)	344 (11.36)	114 (17.27)	117 (14.92)	132 (13.82)	0.0001*
**CCI**						
**Median (IQR)**	0 (0-1)	0 (0-1)	0 (0-1)	0 (0-1)	0 (0-1)	
**Mean (SD)**	0.76 (1.08)	0.71 (1.04)	0.78 (1.07)	0.83 (1.12)	0.85 (1.16)	0.002*
**0-2**	5,042 (92.91)	2,849 (94.09)	607 (91.97)	717 (91.45)	869 (90.99)	0.011*
**3-6**	373 (6.87)	172 (5.68)	52 (7.88)	66 (8.42)	83 (8.69)	
**≥ 7**	12 (0.22)	7 (0.23)	1 (0.15)	1 (0.13)	3 (0.31)	

*AMD* age-related macular degeneration, *CCI* Charlson comorbidity index, *IQR* interquartile range, *LPI* laser peripheral iridotomy, *SD* standard deviation.

Data are presented as no. of patients (%), unless otherwise specified.

*Statistically significant

## Discussion

Our large-scale study, based on a national dataset of adults aged 65 years and older, demonstrated a nationwide increase in cataract surgeries and a concurrent decrease in LPI procedures in Korea between 2016 and 2021. The rates per 100,000 individuals in this age group mirrored this pattern, indicating an inverse temporal association between cataract surgeries and LPI procedures over the study period. When stratified by age, the negative correlation was particularly strong and statistically significant among individuals aged 65–74 years (*r* = −0.943, *P* = 0.005), suggesting that the rise in cataract surgeries in this subgroup was closely aligned with the decline in LPI procedures. However, these opposing trends may also reflect concurrent but independent shifts in clinical practice or other unmeasured factors over time, and thus these findings should be interpreted as correlational rather than causative.

While similar trends have been reported previously [9,11], our study adds greater depth by analyzing nationwide, population-based data that span both pre- and post-COVID-19 periods, with stratification by age and sex. Including the COVID-19 years allowed us to observe how these trends behaved during an abrupt reduction in healthcare utilization, thereby helping to determine whether the observed trends were temporary disruptions or indicative of sustained changes in clinical practice. In 2020, at the onset of the COVID-19 pandemic, both cataract surgeries and LPI procedures decreased, consistent with a report of declines in various ophthalmic procedures during the early pandemic [13]. However, while cataract surgeries rebounded in 2021, LPI procedures continued to decline, suggesting that the continued decline in LPI utilization reflects an ongoing clinical shift rather than a transient pandemic effect. This persistent downward trend—even after cataract surgical volumes recovered—supports the possibility of a broader shift in clinical strategies for managing angle closure. Dense cataracts, particularly nuclear cataracts, can increase lens vault and cause a forward shift of the lens, narrowing the anterior chamber angles and raising the risk of angle closure [14–16]. Cataract surgery may relieve such lens-induced angle narrowing, thereby reducing the need for LPI procedures.

The proportion of females was higher in the LPI group than in the cataract surgery group (73.2% and 58.8%), consistent with previous studies indicating that females are at a higher risk for angle closure [15]. Most patients who underwent cataract surgery received it in clinics (75.5%), while the proportion of those who had the surgery in general hospitals or higher-level facilities was relatively low (16.2%). On the other hand, the proportion of patients who received LPI in clinics was not overwhelmingly high (48.4%), with a relatively high proportion receiving it in general hospitals or higher-level facilities (37.4%). This suggests that patients requiring LPI due to angle closure often have more complex cases, which may lead them to seek treatment at higher-level hospitals.

Most patients who underwent LPI later had cataract surgery (92.11%), with 25% undergoing the procedure within one month (Q1 = 29 days) and 50% within four months (median = 121 days) after LPI. More than half of the patients had cataract surgery within six months (55.8%), and 82.4% within two years. Given the high incidence of cataract surgery following LPI in our cohort—likely reflecting age-related lens changes—this sequence prompts a re-evaluation of LPI’s efficacy and cost-effectiveness as an initial strategy. These findings align with the growing clinical preference for primary lens extraction as a more definitive and economically sustainable strategy.

Several studies—including recent randomized trials such as the Zhongshan Angle-Closure Prevention (ZAP) study [17] and the Singapore Asymptomatic Narrow Angles Laser Iridotomy Study (ANA-LIS) [18]—have highlighted the limitations of LPI, particularly in preventing disease progression, reinforcing the need for more definitive interventions. Other reports also suggest that LPI may accelerate cataract progression and elevate IOP [19], further strengthening the rationale for earlier lens extraction. Early phacoemulsification appears superior to LPI for both IOP control and corneal endothelial protection [20]. A randomized controlled trial (EAGLE study) demonstrated that lens extraction for PAC or PACG is more effective and cost-efficient than LPI combined with topical medical treatment, supporting its role as a first-line treatment option [7]. A study conducted in the United Kingdom also reported that early lens extraction was cost-effective at three years and cost-saving by ten years in patients with PAC or PACG [21].

Although LPI is usually considered a first-line treatment in PACG without cataract [9], clear-lens extraction also lowers IOP and improves visual outcomes in patients with PAC and elevated IOP (≥30 mmHg at diagnosis) or PACG, regardless of the presence of cataract [7]. Cataract surgery benefits not only early PAC patients but also those with severe, end-stage glaucoma [1]. While LPI is sometimes performed to reduce the risk of malignant glaucoma during cataract surgery, it may not consistently prevent and can rarely precipitate this complication [22,23]. Although cataract surgery in acute angle-closure crisis carries higher risks due to shallow anterior chambers and choroidal expansion, early phacoemulsification after initial IOP reduction helps maintain IOP control and reduce medication use [24].

In PAC and PACG, poorly controlled IOP is usually an indication for trabeculectomy, but this surgery is associated with potentially serious complications, such as bleb leak, hypotony, choroidal effusion, choroidal hemorrhage, blebitis, and endophthalmitis [7,25]. In this study, patients who underwent cataract surgery after LPI had a low rate of trabeculectomy (3.6%) and Ahmed valve implantation (1.1%), indicating that IOP was well controlled in most patients who had cataract surgery following LPI.

The limitations of this study include, first, the inability to distinguish specific subtypes within primary angle-closure disease (PACD), such as primary angle-closure suspect (PACS), PAC, and PACG [5]. The only available diagnosis code related to angle closure was H40.2 for PACG, and no separate codes existed for PACS or PAC, making detailed subgroup analysis or PACD-specific cohort construction infeasible. This constraint limits disease-specific trend analysis but does not substantially affect the interpretation of nationwide procedure patterns, which were the primary focus of this study. Second, because this was a descriptive analysis based on population-level procedure data, the observed temporal relationship between increasing cataract surgeries and declining LPI procedures should be interpreted as correlational rather than causal. Important confounders—such as disease severity, socioeconomic factors, and the recent expansion of premium IOL use—could not be adjusted for and may have contributed to the observed trends. Third, the NHID does not include laterality information, preventing identification of whether LPI and cataract surgery were performed in the same eye. This is an inherent constraint of NHID-based research, but it does not materially affect the population-level trends that were the focus of this study.

In conclusion, this nationwide study demonstrated a temporal association and demographic characteristics underlying the recent increase in cataract surgeries and the concurrent decline in LPI procedures in Korea. These findings suggest a paradigm shift in the management of PACD, with growing clinical preference for lens extraction over LPI. Notably, most patients who underwent LPI subsequently received cataract surgery within a relatively short interval, highlighting the need to re-evaluate the efficiency, timing, and role of LPI as an initial intervention in older adults with PACD.
