# Supplementary material for: Declining laser peripheral iridotomy for angle closure alongside rising cataract surgeries: A nationwide cohort study in South Korea
Source: PLoS One. 2026 Feb 20;21(2):e0343427. doi: 10.1371/journal.pone.0343427 (PMC12923034; doi:10.1371/journal.pone.0343427)
Supplement: Attachment — Submitted filename: Response to Reviewers.docx [file pone.0343427.s002.docx]

**Dear Editor-in-Chief**

Along with my coauthors, I would like to express our sincere appreciation to the editors and reviewers for dedicating their valuable time and making insightful contributions to our manuscript. The reviewer's comments were thoughtful and have significantly aided us in improving our manuscript. After carefully reviewing our work, we have implemented appropriate revisions based on the reviewers' suggestions. Below, you will find a point-by-point response to the reviewers' comments.

With many thanks and kind regards,

Sincerely,

Jong Suk Song, MD, PhD

Department of Ophthalmology, Korea University College of Medicine

Department of Ophthalmology, Korea University Guro Hospital

148 Gurodong-ro, Guro-gu, Seoul 08308, Republic of Korea

crisim@korea.ac.kr

Tel: +82-2-2626-1270, Fax: +82-2-583-6521

**Point-by-point response**

**Additional Editor Comments:**

This nationwide, population-based cohort study provides valuable epidemiologic insight into changing clinical practice patterns for primary angle-closure disease in Korea. While the study reports a negative correlation between cataract surgery and LPI volumes, the causal inference remains limited. The analysis is purely descriptive and does not adjust for potential confounders such as disease severity, socioeconomic factors, or the growing use of premium IOLs that may independently increase cataract surgery rates. Please revise the discussion to include the study limitation—specifically, the need to clarify that the findings are correlational and that potential confounding factors were not controlled for.

**Response:** We appreciate the editor’s insightful comment. To clearly state that our findings are correlational rather than causal, we have revised the Limitations section of the Discussion. The revised text explicitly notes that this analysis is descriptive in nature and does not adjust for important confounders, such as disease severity, socioeconomic factors, and trends in premium intraocular lens use. These clarifications have now been incorporated into the Limitations section to more accurately reflect the methodological constraints of our study. The original and revised versions of the relevant section are provided below for clarity.

- Discussion (Line 298):

**Original:** “The limitations of this study include, first, the inability to distinguish specific subtypes within primary angle-closure disease (PACD), such as primary angle-closure suspect (PACS), PAC, and PACG [5]. The only available diagnosis code related to angle closure was H40.2 for PACG, and no separate codes existed for PACS or PAC, making detailed subgroup analysis unfeasible. However, since angle closure is typically coded as PACG in clinical practice, this limitation is unlikely to have significantly impacted our findings. Second, the recent increase in the use of non-reimbursed premium IOLs has accelerated cataract surgeries among patients under 65 years in Korea [10], potentially lowering the incidence of PAC or PACG and reducing the demand for LPI. As our analysis focused on patients aged 65 and older, this trend could not be thoroughly assessed. Nonetheless, the consistent decline in LPI volume within the older population suggests a growing trend toward earlier cataract surgery that may be influencing clinical practice across age groups. Future studies involving younger populations will be needed to better characterize this trend.”

**Revised:** “The limitations of this study include, first, the inability to distinguish specific subtypes within primary angle-closure disease (PACD), such as primary angle-closure suspect (PACS), PAC, and PACG [5]. The only available diagnosis code related to angle closure was H40.2 for PACG, and no separate codes existed for PACS or PAC, making detailed subgroup analysis or PACD-specific cohort construction infeasible. This constraint limits disease-specific trend analysis but does not substantially affect the interpretation of nationwide procedure patterns, which were the primary focus of this study. Second, because this was a descriptive analysis based on population-level procedure data, the observed temporal relationship between increasing cataract surgeries and declining LPI procedures should be interpreted as correlational rather than causal. Important confounders—such as disease severity, socioeconomic factors, and the recent expansion of premium IOL use—could not be adjusted for and may have contributed to the observed trends. Third, the NHID does not include laterality information, preventing identification of whether LPI and cataract surgery were performed in the same eye. This is an inherent constraint of NHID-based research, but it does not materially affect the population-level trends that were the focus of this study.”

**Reviewer #1:**

This study demonstrates that the number of cataract surgeries increased while that of LPI decreased over the 6 years from 2016 to 2021, showing a significant negative correlation between the two based on data from the Korean National Health Information Database. The manuscript is well designed and well written. However, there are some points that need to be revised before it can be considered for publication.

**Comment 1:** The authors abruptly conclude a correlation between cataract surgeries and LPI procedures based on longitudinal observations of these two independent issues. However, they may have changed independently over the years. In fact, the decrease in the number of LPI procedures does not necessarily indicate a decrease in the occurrence of ACD. Accessibility to emergency care at night could also have differed during the study period compared to earlier periods. In addition, in cases of ACD, cataract surgery may have been performed soon after medical treatment for the management of ACD. These points should be considered in the Discussion section.

**Response:** We sincerely thank the reviewer for this valuable comment, which raises important concerns about overinterpreting longitudinal trends as evidence of causality. We fully agree that the observed trends may reflect independent changes in clinical practice patterns, healthcare accessibility (e.g., nighttime emergency care), or other unmeasured systemic factors.

To address this, we have revised the first paragraph of the Discussion to explicitly clarify that the observed inverse trends are correlational and not indicative of a direct causal relationship. We now note that the trends may reflect independent changes over time, and we caution against overinterpretation.

In addition, we acknowledge the reviewer’s point that in some acute angle-closure cases, early cataract surgery may have been performed soon after medical management without preceding LPI. This possibility aligns with our broader interpretation that clinical strategies may be evolving toward early lens extraction. To reflect this perspective, we have incorporated a statement into the Discussion noting that the high incidence of cataract surgery following LPI in our cohort supports re-evaluating LPI as an initial intervention in older adults.

The following revisions were made:

- Discussion (Line 231):

**Original:** “When analyzed by age, this negative correlation was particularly strong and statistically significant among subjects aged 65 to 74 years (*r* = -0.943, *P* = 0.005), indicating that the rise in cataract surgeries in this relatively younger age group had a substantial impact on the decrease in LPI procedures.”

**Revised:** “When stratified by age, the negative correlation was particularly strong and statistically significant among individuals aged 65 to 74 years (r = -0.943, P = 0.005), suggesting that the rise in cataract surgeries in this subgroup was closely aligned with the decline in LPI procedures.”

- Discussion (Line 234):

**Added:** “However, these opposing trends may also reflect concurrent but independent shifts in clinical practice or other unmeasured factors over time, and thus these findings should be interpreted as correlational rather than causative.”

- Discussion (Line 265):

**Added:** “Given the high incidence of cataract surgery following LPI in our cohort—likely reflecting age-related lens changes—this sequence prompts a re-evaluation of LPI’s efficacy and cost-effectiveness as an initial strategy.”

We also revised the Abstract to reflect a more cautious interpretation of this association. The concluding sentence was updated to avoid implying causality and to align with the reviewer’s concern about overinterpretation:

- Abstract (Line 38):

**Original:** “Notably, most patients who underwent LPI subsequently received cataract surgery within a relatively short interval, reflecting an evolving trend in the clinical management of PACD that may increasingly favor lens extraction over time.”

**Revised:** “Notably, most patients who underwent LPI subsequently received cataract surgery within a relatively short interval, suggesting a possible clinical shift toward earlier lens extraction in the management of angle closure.”

**Comment 2:** The authors mention that the significance of this study lies in the inclusion of the COVID-19 period. However, it is not clear why this is particularly meaningful for the present study. Although the data collection period included the COVID-19 years, the study also includes data from 2016, which was long before COVID-19 became a major issue. The authors should clarify the importance of this point.

**Response:** Thank you for this insightful comment. We have clarified why inclusion of both pre- and post-COVID-19 years is important for interpreting the observed trends. The COVID-19 period represents an abrupt, nationwide reduction in healthcare utilization, functioning as a natural stress test for longitudinal procedure trends. Including these years allowed us to assess whether the decrease in LPI and the rise in cataract surgery represented temporary pandemic-related fluctuations or a persistent long-term shift in clinical practice.

We have revised the Discussion accordingly to explicitly state this rationale.

- Discussion (Line 237):

**Original:** “The increase in cataract surgeries and the corresponding decrease in LPI procedures have been documented in earlier studies [9, 11]. However, our study provides a more comprehensive and updated perspective, as it is the first to analyze nationwide, population-based data across both pre- and post-COVID-19 periods, with analyses stratified by age and sex. This observed shift may be linked to anatomical and clinical factors discussed in prior research. Dense cataracts, particularly nuclear cataracts, can increase lens vault and cause a forward shift of the lens, which may narrow the anterior chamber angles and raise the risk of angle closure [13-15]. Therefore, cataract surgery can relieve angle closure caused by the lens, leading to a decrease in LPI procedures. In 2020, at the onset of the COVID-19 pandemic, both cataract surgeries and LPI procedures decreased, consistent with a report of declines in various ophthalmic procedures during the early pandemic [16]. However, while cataract surgeries rebounded in 2021, LPI procedures continued to decline, suggesting that the decrease in LPI was not solely attributable to the pandemic but may also reflect an ongoing trend toward earlier cataract surgery.”

**Revised:** “While similar trends have been reported previously [9, 11], our study adds greater depth by analyzing nationwide, population-based data that span both pre- and post-COVID-19 periods, with stratification by age and sex. Including the COVID-19 years allowed us to observe how these trends behaved during an abrupt reduction in healthcare utilization, thereby helping to determine whether the observed trends were temporary disruptions or indicative of sustained changes in clinical practice. In 2020, at the onset of the COVID-19 pandemic, both cataract surgeries and LPI procedures decreased, consistent with a report of declines in various ophthalmic procedures during the early pandemic [13]. However, while cataract surgeries rebounded in 2021, LPI procedures continued to decline, suggesting that the continued decline in LPI utilization reflects an ongoing clinical shift rather than a transient pandemic effect. This persistent downward trend—even after cataract surgical volumes recovered—supports the possibility of a broader shift in clinical strategies for managing angle closure. Dense cataracts, particularly nuclear cataracts, can increase lens vault and cause a forward shift of the lens, narrowing the anterior chamber angles and raising the risk of angle closure [14–16]. Cataract surgery may relieve such lens-induced angle narrowing, thereby reducing the need for LPI procedures.”

**Reviewer #2:**

Woojin Kim et al. reported “Declining laser peripheral iridotomy for angle closure alongside rising cataract surgeries: A nationwide cohort study in South Korea.” The study showed a temporal association and demographic characteristics underlying the recent increase in cataract surgeries and the concurrent decline in LPI procedures in Korea. Notably, most patients who underwent LPI subsequently received cataract surgery within a relatively short interval, reflecting an evolving trend in the clinical management of PACD that may increasingly favor lens extraction over time.

It is an interesting study; however, the study still needs to address some issues.

**Comment 1:** A person has two eyes. Please clarify how the codes distinguished between the right and left eyes.

**Response:** We appreciate the reviewer’s thoughtful comment regarding laterality. As the Korean National Health Information Database (NHID) does not specify laterality, all analyses were conducted at the person-level.

While this precludes eye-specific associations, we believe it does not materially affect the interpretation of our findings for the following reasons. First, primary angle-closure disease (PACD) has a strong bilateral tendency, and LPI is frequently performed in both eyes, either simultaneously or sequentially. Similarly, cataract surgery in older adults is commonly bilateral, typically performed within a short interval.

Therefore, the person-level pattern of high cataract surgery rates and short intervals after LPI aligns with real-world clinical practice. Moreover, the primary objective of our study was to evaluate population-level trends over time, not eye-specific outcomes. Person-level analysis is thus appropriate and consistent with prior NHID-based studies.

We have clarified this limitation in the Methods and Discussion sections:

- Methods (Line 106):

**Added:** “Because the NHID does not provide laterality information, all analyses were performed at the person-level, and eye-specific relationships could not be determined.”

- Discussion (Line 309):

**Added:** “Third, the NHID does not include laterality information, preventing identification of whether LPI and cataract surgery were performed in the same eye. This is an inherent constraint of NHID-based research, but it does not materially affect the population-level trends that were the focus of this study.”

**Comment 2:** The authors mentioned “suggesting that rising cataract surgeries likely contributed to the decline in LPI use.” However, since the analysis could not distinguish between the two eyes of a single patient and was based only on incidence data, it seems difficult to draw this causal conclusion.

**Response:** Thank you for this valuable comment. We agree that the original phrase (“likely contributed”) may have implied a causal relationship, which cannot be supported by our incidence-based, person-level analysis that lacks laterality information. Our intent was to describe a temporal association, not causality.

Accordingly, we have revised the sentence in the Discussion to reflect associative—rather than causal—language, in line with the descriptive nature of our study.

- Discussion (Line 229):

**Original:** “The rates per 100,000 population mirrored this pattern, suggesting that rising cataract surgeries likely contributed to the decline in LPI use.”

**Revised:** “The rates per 100,000 individuals in this age group mirrored this pattern, indicating an inverse temporal association between cataract surgeries and LPI procedures over the study period.”

**Comment 3:** The statement “The annual number of patients who underwent cataract surgery or LPI for PACG was analyzed, along with the rate of these procedures per 100,000 population” (lines 112-113) — does this refer to the rate among the entire Korean population, or only among those aged 65 years and older?

**Response:** Thank you for raising this important point. The rates per 100,000 population in our study were calculated using the annual population of adults aged 65 years and older, not the entire Korean population. Because our cohort was restricted to individuals aged 65 and above, we used the corresponding age-specific population from Statistics Korea to ensure appropriate standardization.

We revised the Methods and Results sections accordingly.

- Methods (Line 113):

**Original:** “The annual number of patients who underwent cataract surgery or LPI for PACG was analyzed, along with the rate of these procedures per 100,000 population.”

**Revised:** “The annual number of patients who underwent cataract surgery or LPI for PACG was analyzed, along with the rate of these procedures per 100,000 persons aged 65 years or older.”

- Methods (Line 131):

**Original:** “For the calculation of the rate per 100,000 population, the annual population census data from Statistics Korea was used.”

**Revised:** “For the calculation of the rate per 100,000 population, we used the annual population of adults aged 65 years and older obtained from Statistics Korea.”

- Results (Line 156):

**Original:** “Similarly, the number of cataract surgeries per 100,000 population rose from 2,621 in 2017 to 2,869 in 2019, declined to 2,477 in 2020, and then increased again to 2,544 in 2021 (detailed annual rates: [2016: 2,672], [2017: 2,621], [2018: 2,633], [2019: 2,869], [2020: 2,477], [2021: 2,544]).”

**Revised:** “Similarly, the number of cataract surgeries per 100,000 individuals aged 65 years and older rose from 2,621 in 2017 to 2,869 in 2019, declined to 2,477 in 2020, and then increased again to 2,544 in 2021 (detailed annual rates: [2016: 2,672], [2017: 2,621], [2018: 2,633], [2019: 2,869], [2020: 2,477], [2021: 2,544]).”

- Results (Line 161):

**Original:** “Likewise, the number of LPI procedures per 100,000 population decreased consistently from 13.27 in 2017 to 5.62 in 2021 (annual rates: [2016: 12.82], [2017: 13.27], [2018: 12.2], [2019: 10.24], [2020: 7.62], [2021: 5.62]).”

**Revised:** “Likewise, the number of LPI procedures per 100,000 individuals aged 65 years and older decreased consistently from 13.27 in 2017 to 5.62 in 2021 (annual rates: [2016: 12.82], [2017: 13.27], [2018: 12.2], [2019: 10.24], [2020: 7.62], [2021: 5.62]).”

**Comment 4:** The increase in cataract surgeries and the decrease in LPI might have been affected by separate factors. To clarify this relationship, rather than analyzing only patients who underwent cataract surgery after LPI, it would be more appropriate to analyze, as in the studies cited by the authors (PMID: 24193602, 36252921), the annual trend of cataract surgery and LPI among patients diagnosed with PAC or PACG.

**Response:** Thank you for this constructive comment. We agree that analyzing procedure trends specifically within a PAC or PACG cohort would provide disease-specific insights, as demonstrated in the cited studies (PMID: 24193602, 36252921). However, the aim of our study was different: rather than characterizing trends within a defined disease cohort, our objective was to evaluate nationwide changes in the overall utilization of cataract surgery and LPI and to describe how these two procedure patterns have shifted over time in clinical practice.

In Korea, cataract surgery volume has been increasing broadly across the population due to multiple factors—including advances in surgical safety, growing patient expectations, and the widespread availability of diverse IOL options. Many individuals who might previously have undergone LPI for narrow angles now receive cataract surgery directly, even before a formal diagnosis of PAC or PACG is assigned. As a result, restricting the analysis to patients with a recorded PAC or PACG diagnosis (H40.2) would omit a substantial proportion of individuals for whom cataract surgery is now frequently preferred in clinical situations where LPI might once have been considered, reflecting evolving management strategies. Moreover, as noted in the limitations section, the NHID only includes a single diagnostic code (H40.2) for PACG, limiting the ability to construct a detailed PACD-specific cohort.

For these reasons, analyzing nationwide procedure trends without restricting to PACD-coded patients was methodologically appropriate for our research question.

**Comment 5:** Systemic comorbidity was assessed using the Charlson comorbidity index (CCI). Please describe briefly what this index represents for readers unfamiliar with it.

**Response:** We agree that a brief explanation of the Charlson Comorbidity Index (CCI) would help readers unfamiliar with the measure. We have added a concise description to the Methods section to clarify that the CCI is a validated weighted score reflecting a patient’s overall burden of systemic disease.

- Methods (Line 119):

**Original:** “Systemic comorbidity was assessed using the Charlson comorbidity index (CCI), based on the methodology described by Charlson et al [12].”

**Revised:** “Systemic comorbidity was assessed using the Charlson Comorbidity Index (CCI), a validated weighted score summarizing a patient’s overall systemic disease burden based on predefined medical conditions [12].”

**Comment 6:** Were there any age differences between patients who underwent cataract surgery after LPI and patients who underwent LPI alone?

Such a difference could have influenced the results related to AMD and CCI scores. If there was an age difference, statistical adjustment would be required.

**Response:** We examined whether age differences existed between patients who underwent cataract surgery after LPI and those who underwent LPI alone. As shown in Tables 2 and 3, patients who subsequently underwent cataract surgery after LPI had a mean age of 74.20 ± 5.64 years, whereas the overall LPI cohort had a mean age of 73.30 ± 5.75 years.

The mean difference of approximately 0.9 years is minimal, and the median and interquartile range values were also nearly identical (median 74 vs. 73 years; IQR 70–78 vs. 69–77 years), indicating no meaningful age difference between the groups. Because the age profiles were essentially the same, age is unlikely to account for the observed differences in AMD prevalence or CCI scores, and additional statistical adjustment for age was not required.

A clarifying sentence has been added to the Results section.

- Results (Line 204):

**Added:** “Age distributions were comparable between patients who underwent cataract surgery after LPI and those who underwent LPI alone (mean 74.20 ± 5.64 vs. 73.30 ± 5.75 years).”

**Comment 7:** In Table 3, the meaning of the “P value for trend” is not clearly explained in the Results section. Please clarify what this P value indicates and why it was analyzed.

**Response:** Thank you for the helpful comment. We agree that the original label “P value for trend” was not appropriate, as the comparisons in Table 3 involved four discrete interval groups without an assumption of monotonic trend. Accordingly, we revised the table heading to “P value.” A clarifying sentence has also been added to the Results section to indicate that these values represent statistical comparisons across the groups, but the differences were small and not clinically meaningful.

- Results (Line 216):

**Added:** “P values were calculated to assess whether patient characteristics varied across the four interval groups. While some comparisons were statistically significant, the differences were small and not considered clinically meaningful.”

- Table 3 heading:

Revised from “*P* value for trend” → “*P* value”

**Comment 8:** The following statement may be unnecessary: “Additionally, when analyzed by gender, negative correlations were observed in both females and males, with a stronger—though still non-significant—association in females (r = −0.771, P = 0.072; males: r = −0.543, P = 0.266).”

Since these results are not statistically significant, it would be better to omit this part.

**Response:** We agree that this sentence was not essential for interpretation. Since the gender-stratified correlations did not reach statistical significance, we have removed this statement from the manuscript to keep the Discussion concise and focused. This revision also helps maintain clarity by avoiding overinterpretation of non-significant subgroup results.

**Comment 9:** The authors mentioned an “ongoing trend toward earlier cataract surgery” (line 240). However, in Table 2, both the median and mean ages show little change over time, and the proportion of patients aged 65–74 years even tends to decrease. To substantiate this claim, data analysis should include a younger population (e.g., patients in their 50s), not only those aged 65 years and above.

**Response:** Thank you for this thoughtful comment. We agree that the phrase “ongoing trend toward earlier cataract surgery” may have implied a shift into younger age groups that are not captured in our dataset, which included only individuals aged 65 years and older. Accordingly, we revised the wording to avoid overinterpretation beyond the available age range.

We also note that, as shown in Table 1, which presents the characteristics of cataract surgery recipients, the proportion of patients aged 65–74 years increased steadily from 52.71% in 2016 to 58.44% in 2021, while the mean age declined from 74.45 to 73.75 years. These findings suggest a gradual shift toward performing cataract surgery at slightly younger ages within the older adult cohort. To reflect this nuance more accurately, we revised the Discussion as follows:

- Discussion (Line 244):

**Original:** “However, while cataract surgeries rebounded in 2021, LPI procedures continued to decline, suggesting that the decrease in LPI was not solely attributable to the pandemic but may also reflect an ongoing trend toward earlier cataract surgery.”

**Revised:** “However, while cataract surgeries rebounded in 2021, LPI procedures continued to decline, suggesting that the continued decline in LPI utilization reflects an ongoing clinical shift rather than a transient pandemic effect.”
