# Supplementary material for: Declining laser peripheral iridotomy for angle closure alongside rising cataract surgeries: A nationwide cohort study in South Korea
Source: PLoS One. 2026 Feb 20;21(2):e0343427. doi: 10.1371/journal.pone.0343427 (PMC12923034; doi:10.1371/journal.pone.0343427)
Supplement: Peer review history

# Peer review: Decision Letter 0

**DOI:** 10.1371/journal.pone.0343427.r001

**Authors:** Daniel Duck-Jin Hwang

12 Nov 2025

Dear Dr. Song,

Thank you for submitting your manuscript to PLOS ONE. After careful consideration, we feel that it has merit but does not fully meet PLOS ONE’s publication criteria as it currently stands. Therefore, we invite you to submit a revised version of the manuscript that addresses the points raised during the review process.

Please submit your revised manuscript by Dec 27 2025 11:59PM. If you will need more time than this to complete your revisions, please reply to this message or contact the journal office at plosone@plos.org . When you're ready to submit your revision, log on to https://www.editorialmanager.com/pone/ and select the 'Submissions Needing Revision' folder to locate your manuscript file.

- A rebuttal letter that responds to each point raised by the academic editor and reviewer(s). You should upload this letter as a separate file labeled 'Response to Reviewers'.
- A marked-up copy of your manuscript that highlights changes made to the original version. You should upload this as a separate file labeled 'Revised Manuscript with Track Changes'.
- An unmarked version of your revised paper without tracked changes. You should upload this as a separate file labeled 'Manuscript'.

If you would like to make changes to your financial disclosure, please include your updated statement in your cover letter. Guidelines for resubmitting your figure files are available below the reviewer comments at the end of this letter.

If applicable, we recommend that you deposit your laboratory protocols in protocols.io to enhance the reproducibility of your results. Protocols.io assigns your protocol its own identifier (DOI) so that it can be cited independently in the future. For instructions see: https://journals.plos.org/plosone/s/submission-guidelines#loc-laboratory-protocols . Additionally, PLOS ONE offers an option for publishing peer-reviewed Lab Protocol articles, which describe protocols hosted on protocols.io. Read more information on sharing protocols at https://plos.org/protocols?utm_medium=editorial-email&utm_source=authorletters&utm_campaign=protocols .

We look forward to receiving your revised manuscript.

Kind regards,

Daniel Duck-Jin Hwang

Academic Editor

PLOS ONE

Journal Requirements:

When submitting your revision, we need you to address these additional requirements.

1. Please ensure that your manuscript meets PLOS ONE's style requirements, including those for file naming. The PLOS ONE style templates can be found at

https://journals.plos.org/plosone/s/file?id=wjVg/PLOSOne_formatting_sample_main_body.pdf and

https://journals.plos.org/plosone/s/file?id=ba62/PLOSOne_formatting_sample_title_authors_affiliations.pdf

2. Please note that PLOS One has specific guidelines on code sharing for submissions in which author-generated code underpins the findings in the manuscript. In these cases, we expect all author-generated code to be made available without restrictions upon publication of the work. Please review our guidelines at https://journals.plos.org/plosone/s/materials-and-software-sharing#loc-sharing-code and ensure that your code is shared in a way that follows best practice and facilitates reproducibility and reuse.

3. Please include your tables as part of your main manuscript and remove the individual files. Please note that supplementary tables (should remain/ be uploaded) as separate "supporting information" files

4. We note that you have indicated that there are restrictions to data sharing for this study. For studies involving human research participant data or other sensitive data, we encourage authors to share de-identified or anonymized data. However, when data cannot be publicly shared for ethical reasons, we allow authors to make their data sets available upon request. For information on unacceptable data access restrictions, please see http://journals.plos.org/plosone/s/data-availability#loc-unacceptable-data-access-restrictions.

Before we proceed with your manuscript, please address the following prompts:

a) If there are ethical or legal restrictions on sharing a de-identified data set, please explain them in detail (e.g., data contain potentially identifying or sensitive patient information, data are owned by a third-party organization, etc.) and who has imposed them (e.g., a Research Ethics Committee or Institutional Review Board, etc.). Please also provide contact information for a data access committee, ethics committee, or other institutional body to which data requests may be sent.

b) If there are no restrictions, please upload the minimal anonymized data set necessary to replicate your study findings to a stable, public repository and provide us with the relevant URLs, DOIs, or accession numbers. Please see http://www.bmj.com/content/340/bmj.c181.long for guidelines on how to de-identify and prepare clinical data for publication. For a list of recommended repositories, please see https://journals.plos.org/plosone/s/recommended-repositories. You also have the option of uploading the data as Supporting Information files, but we would recommend depositing data directly to a data repository if possible.

Please update your Data Availability statement in the submission form accordingly.

5. If the reviewer comments include a recommendation to cite specific previously published works, please review and evaluate these publications to determine whether they are relevant and should be cited. There is no requirement to cite these works unless the editor has indicated otherwise.

Additional Editor Comments:

This nationwide, population-based cohort study provides valuable epidemiologic insight into changing clinical practice patterns for primary angle-closure disease in Korea. While the study reports a negative correlation between cataract surgery and LPI volumes, the causal inference remains limited. The analysis is purely descriptive and does not adjust for potential confounders such as disease severity, socioeconomic factors, or the growing use of premium IOLs that may independently increase cataract surgery rates. Please revise the discussion to include the study limitation—specifically, the need to clarify that the findings are correlational and that potential confounding factors were not controlled for.

[Note: HTML markup is below. Please do not edit.]

Reviewers' comments:

Reviewer's Responses to Questions

**Comments to the Author**

1. Is the manuscript technically sound, and do the data support the conclusions?

Reviewer #1: Yes

Reviewer #2: Partly

**********

2. Has the statistical analysis been performed appropriately and rigorously?

Reviewer #1: Yes

Reviewer #2: Yes

**********

3. Have the authors made all data underlying the findings in their manuscript fully available?

The PLOS Data policy

Reviewer #1: Yes

Reviewer #2: Yes

**********

4. Is the manuscript presented in an intelligible fashion and written in standard English?

Reviewer #1: Yes

Reviewer #2: Yes

**********

Reviewer #1: This study demonstrates that the number of cataract surgeries increased while that of LPI decreased over the 6 years from 2016 to 2021, showing a significant negative correlation between the two based on data from the Korean National Health Information Database. The manuscript is well designed and well written. However, there are some points that need to be revised before it can be considered for publication.

1. The authors abruptly conclude a correlation between cataract surgeries and LPI procedures based on longitudinal observations of these two independent issues. However, they may have changed independently over the years. In fact, the decrease in the number of LPI procedures does not necessarily indicate a decrease in the occurrence of ACD. Accessibility to emergency care at night could also have differed during the study period compared to earlier periods. In addition, in cases of ACD, cataract surgery may have been performed soon after medical treatment for the management of ACD. These points should be considered in the Discussion section.

2. The authors mention that the significance of this study lies in the inclusion of the COVID-19 period. However, it is not clear why this is particularly meaningful for the present study. Although the data collection period included the COVID-19 years, the study also includes data from 2016, which was long before COVID-19 became a major issue. The authors should clarify the importance of this point.

Reviewer #2: Woojin Kim et al. reported “Declining laser peripheral iridotomy for angle closure alongside rising cataract surgeries: A nationwide cohort study in South Korea.” The study showed a temporal association and demographic characteristics underlying the recent increase in cataract surgeries and the concurrent decline in LPI procedures in Korea. Notably, most patients who underwent LPI subsequently received cataract surgery within a relatively short interval, reflecting an evolving trend in the clinical management of PACD that may increasingly favor lens extraction over time.

It is an interesting study; however, the study still needs to address some issues.

- A person has two eyes. Please clarify how the codes distinguished between the right and left eyes.

- The authors mentioned “suggesting that rising cataract surgeries likely contributed to the decline in LPI use.” However, since the analysis could not distinguish between the two eyes of a single patient and was based only on incidence data, it seems difficult to draw this causal conclusion.

- The statement “The annual number of patients who underwent cataract surgery or LPI for PACG was analyzed, along with the rate of these procedures per 100,000 population” (lines 112-113) — does this refer to the rate among the entire Korean population, or only among those aged 65 years and older?

- The increase in cataract surgeries and the decrease in LPI might have been affected by separate factors. To clarify this relationship, rather than analyzing only patients who underwent cataract surgery after LPI, it would be more appropriate to analyze, as in the studies cited by the authors (PMID: 24193602, 36252921), the annual trend of cataract surgery and LPI among patients diagnosed with PAC or PACG.

- Systemic comorbidity was assessed using the Charlson comorbidity index (CCI). Please describe briefly what this index represents for readers unfamiliar with it.

- Were there any age differences between patients who underwent cataract surgery after LPI and patients who underwent LPI alone?

Such a difference could have influenced the results related to AMD and CCI scores. If there was an age difference, statistical adjustment would be required.

- In Table 3, the meaning of the “P value for trend” is not clearly explained in the Results section. Please clarify what this P value indicates and why it was analyzed.

- The following statement may be unnecessary: “Additionally, when analyzed by gender, negative correlations were observed in both females and males, with a stronger—though still non-significant—association in females (r = −0.771, P = 0.072; males: r = −0.543, P = 0.266).”

Since these results are not statistically significant, it would be better to omit this part.

- The authors mentioned an “ongoing trend toward earlier cataract surgery” (line 240). However, in Table 2, both the median and mean ages show little change over time, and the proportion of patients aged 65–74 years even tends to decrease. To substantiate this claim, data analysis should include a younger population (e.g., patients in their 50s), not only those aged 65 years and above.

**********

what does this mean? ). If published, this will include your full peer review and any attached files.

If you choose “no”, your identity will remain anonymous but your review may still be made public.

**Do you want your identity to be public for this peer review?** For information about this choice, including consent withdrawal, please see our Privacy Policy

Reviewer #1: No

Reviewer #2: No

**********

[NOTE: If reviewer comments were submitted as an attachment file, they will be attached to this email and accessible via the submission site. Please log into your account, locate the manuscript record, and check for the action link "View Attachments". If this link does not appear, there are no attachment files.]

To ensure your figures meet our technical requirements, please review our figure guidelines: https://journals.plos.org/plosone/s/figures

You may also use PLOS’s free figure tool, NAAS, to help you prepare publication quality figures: https://journals.plos.org/plosone/s/figures#loc-tools-for-figure-preparation.

NAAS will assess whether your figures meet our technical requirements by comparing each figure against our figure specifications.

# Author comment: Author response to Decision Letter 1

**DOI:** 10.1371/journal.pone.0343427.r002

10 Dec 2025

Point-by-point response

Additional Editor Comments:

This nationwide, population-based cohort study provides valuable epidemiologic insight into changing clinical practice patterns for primary angle-closure disease in Korea. While the study reports a negative correlation between cataract surgery and LPI volumes, the causal inference remains limited. The analysis is purely descriptive and does not adjust for potential confounders such as disease severity, socioeconomic factors, or the growing use of premium IOLs that may independently increase cataract surgery rates. Please revise the discussion to include the study limitation—specifically, the need to clarify that the findings are correlational and that potential confounding factors were not controlled for.

Response: We appreciate the editor’s insightful comment. To clearly state that our findings are correlational rather than causal, we have revised the Limitations section of the Discussion. The revised text explicitly notes that this analysis is descriptive in nature and does not adjust for important confounders, such as disease severity, socioeconomic factors, and trends in premium intraocular lens use. These clarifications have now been incorporated into the Limitations section to more accurately reflect the methodological constraints of our study. The original and revised versions of the relevant section are provided below for clarity.

• Discussion (Line 298):

Original: “The limitations of this study include, first, the inability to distinguish specific subtypes within primary angle-closure disease (PACD), such as primary angle-closure suspect (PACS), PAC, and PACG [5]. The only available diagnosis code related to angle closure was H40.2 for PACG, and no separate codes existed for PACS or PAC, making detailed subgroup analysis unfeasible. However, since angle closure is typically coded as PACG in clinical practice, this limitation is unlikely to have significantly impacted our findings. Second, the recent increase in the use of non-reimbursed premium IOLs has accelerated cataract surgeries among patients under 65 years in Korea [10], potentially lowering the incidence of PAC or PACG and reducing the demand for LPI. As our analysis focused on patients aged 65 and older, this trend could not be thoroughly assessed. Nonetheless, the consistent decline in LPI volume within the older population suggests a growing trend toward earlier cataract surgery that may be influencing clinical practice across age groups. Future studies involving younger populations will be needed to better characterize this trend.”

Revised: “The limitations of this study include, first, the inability to distinguish specific subtypes within primary angle-closure disease (PACD), such as primary angle-closure suspect (PACS), PAC, and PACG [5]. The only available diagnosis code related to angle closure was H40.2 for PACG, and no separate codes existed for PACS or PAC, making detailed subgroup analysis or PACD-specific cohort construction infeasible. This constraint limits disease-specific trend analysis but does not substantially affect the interpretation of nationwide procedure patterns, which were the primary focus of this study. Second, because this was a descriptive analysis based on population-level procedure data, the observed temporal relationship between increasing cataract surgeries and declining LPI procedures should be interpreted as correlational rather than causal. Important confounders—such as disease severity, socioeconomic factors, and the recent expansion of premium IOL use—could not be adjusted for and may have contributed to the observed trends. Third, the NHID does not include laterality information, preventing identification of whether LPI and cataract surgery were performed in the same eye. This is an inherent constraint of NHID-based research, but it does not materially affect the population-level trends that were the focus of this study.”

Reviewer #1:

This study demonstrates that the number of cataract surgeries increased while that of LPI decreased over the 6 years from 2016 to 2021, showing a significant negative correlation between the two based on data from the Korean National Health Information Database. The manuscript is well designed and well written. However, there are some points that need to be revised before it can be considered for publication.

Comment 1: The authors abruptly conclude a correlation between cataract surgeries and LPI procedures based on longitudinal observations of these two independent issues. However, they may have changed independently over the years. In fact, the decrease in the number of LPI procedures does not necessarily indicate a decrease in the occurrence of ACD. Accessibility to emergency care at night could also have differed during the study period compared to earlier periods. In addition, in cases of ACD, cataract surgery may have been performed soon after medical treatment for the management of ACD. These points should be considered in the Discussion section.

Response: We sincerely thank the reviewer for this valuable comment, which raises important concerns about overinterpreting longitudinal trends as evidence of causality. We fully agree that the observed trends may reflect independent changes in clinical practice patterns, healthcare accessibility (e.g., nighttime emergency care), or other unmeasured systemic factors.

To address this, we have revised the first paragraph of the Discussion to explicitly clarify that the observed inverse trends are correlational and not indicative of a direct causal relationship. We now note that the trends may reflect independent changes over time, and we caution against overinterpretation.

In addition, we acknowledge the reviewer’s point that in some acute angle-closure cases, early cataract surgery may have been performed soon after medical management without preceding LPI. This possibility aligns with our broader interpretation that clinical strategies may be evolving toward early lens extraction. To reflect this perspective, we have incorporated a statement into the Discussion noting that the high incidence of cataract surgery following LPI in our cohort supports re-evaluating LPI as an initial intervention in older adults.

The following revisions were made:

• Discussion (Line 231):

Original: “When analyzed by age, this negative correlation was particularly strong and statistically significant among subjects aged 65 to 74 years (r = -0.943, P = 0.005), indicating that the rise in cataract surgeries in this relatively younger age group had a substantial impact on the decrease in LPI procedures.”

Revised: “When stratified by age, the negative correlation was particularly strong and statistically significant among individuals aged 65 to 74 years (r = -0.943, P = 0.005), suggesting that the rise in cataract surgeries in this subgroup was closely aligned with the decline in LPI procedures.”

• Discussion (Line 234):

Added: “However, these opposing trends may also reflect concurrent but independent shifts in clinical practice or other unmeasured factors over time, and thus these findings should be interpreted as correlational rather than causative.”

• Discussion (Line 265):

Added: “Given the high incidence of cataract surgery following LPI in our cohort—likely reflecting age-related lens changes—this sequence prompts a re-evaluation of LPI’s efficacy and cost-effectiveness as an initial strategy.”

We also revised the Abstract to reflect a more cautious interpretation of this association. The concluding sentence was updated to avoid implying causality and to align with the reviewer’s concern about overinterpretation:

• Abstract (Line 38):

Original: “Notably, most patients who underwent LPI subsequently received cataract surgery within a relatively short interval, reflecting an evolving trend in the clinical management of PACD that may increasingly favor lens extraction over time.”

Revised: “Notably, most patients who underwent LPI subsequently received cataract surgery within a relatively short interval, suggesting a possible clinical shift toward earlier lens extraction in the management of angle closure.”

Comment 2: The authors mention that the significance of this study lies in the inclusion of the COVID-19 period. However, it is not clear why this is particularly meaningful for the present study. Although the data collection period included the COVID-19 years, the study also includes data from 2016, which was long before COVID-19 became a major issue. The authors should clarify the importance of this point.

Response: Thank you for this insightful comment. We have clarified why inclusion of both pre- and post-COVID-19 years is important for interpreting the observed trends. The COVID-19 period represents an abrupt, nationwide reduction in healthcare utilization, functioning as a natural stress test for longitudinal procedure trends. Including these years allowed us to assess whether the decrease in LPI and the rise in cataract surgery represented temporary pandemic-related fluctuations or a persistent long-term shift in clinical practice.

We have revised the Discussion accordingly to explicitly state this rationale.

• Discussion (Line 237):

Original: “The increase in cataract surgeries and the corresponding decrease in LPI procedures have been documented in earlier studies [9, 11]. However, our study provides a more comprehensive and updated perspective, as it is the first to analyze nationwide, population-based data across both pre- and post-COVID-19 periods, with analyses stratified by age and sex. This observed shift may be linked to anatomical and clinical factors discussed in prior research. Dense cataracts, particularly nuclear cataracts, can increase lens vault and cause a forward shift of the lens, which may narrow the anterior chamber angles and raise the risk of angle closure [13-15]. Therefore, cataract surgery can relieve angle closure caused by the lens, leading to a decrease in LPI procedures. In 2020, at the onset of the COVID-19 pandemic, both cataract surgeries and LPI procedures decreased, consistent with a report of declines in various ophthalmic procedures during the early pandemic [16]. However, while cataract surgeries rebounded in 2021, LPI procedures continued to decline, suggesting that the decrease in LPI was not solely attributable to the pandemic but may also reflect an ongoing trend toward earlier cataract surgery.”

Revised: “While similar trends have been reported previously [9, 11], our study adds greater depth by analyzing nationwide, population-based data that span both pre- and post-COVID-19 periods, with stratification by age and sex. Including the COVID-19 years allowed us to observe how these trends behaved during an abrupt reduction in healthcare utilization, thereby helping to determine whether the observed trends were temporary disruptions or indicative of sustained changes in clinical practice. In 2020, at the onset of the COVID-19 pandemic, both cataract surgeries and LPI procedures decreased, consistent with a report of declines in various ophthalmic procedures during the early pandemic [13]. However, while cataract surgeries rebounded in 2021, LPI procedures continued to decline, suggesting that the continued decline in LPI utilization reflects an ongoing clinical shift rather than a transient pandemic effect. This persistent downward trend—even after cataract surgical volumes recovered—supports the possibility of a broader shift in clinical strategies for managing angle closure. Dense cataracts, particularly nuclear cataracts, can increase lens vault and cause a forward shift of the lens, narrowing the anterior chamber angles and raising the risk of angle closure [14–16]. Cataract surgery may relieve such lens-induced angle narrowing, thereby reducing the need for LPI procedures.”

Reviewer #2:

Woojin Kim et al. reported “Declining laser peripheral iridotomy for angle closure alongside rising cataract surgeries: A nationwide cohort study in South Korea.” The study showed a temporal association and demographic characteristics underlying the recent increase in cataract surgeries and the concurrent decline in LPI procedures in Korea. Notably, most patients who underwent LPI subsequently received cataract surgery within a relatively short interval, reflecting an evolving trend in the clinical management of PACD that may increasingly favor lens extraction over time.

It is an interesting study; however, the study still needs to address some issues.

Comment 1: A person has two eyes. Please clarify how the codes distinguished between the right and left eyes.

Response: We appreciate the reviewer’s thoughtful comment regarding laterality. As the Korean National Health Information Database (NHID) does not specify laterality, all analyses were conducted at the person-level.

While this precludes eye-specific associations, we believe it does not materially affect the interpretation of our findings for the following reasons. First, primary angle-closure disease (PACD) has a strong bilateral tendency, and LPI is frequently performed in both eyes, either simultaneously or sequentially. Similarly, cataract surgery in older adults is commonly bilateral, typically performed within a short interval.

Therefore, the person-level pattern of high cataract surgery rates and short intervals after LPI aligns with real-world clinical practice. Moreover, the primary objective of our study was to evaluate population-level trends over time, not eye-specific outcomes. Person-level analysis is thus appropriate and consistent with prior NHID-based studies.

We have clarified this limitation in the Methods and Discussion sections:

• Methods (Line 106):

Added: “Because the NHID does not provide laterality information, all analyses were performed at the person-level, and eye-specific relationships could not be determined.”

• Discussion (Line 309):

Added: “Third, the NHID does not include laterality information, preventing identification of whether LPI and cataract surgery were performed in the same eye. This is an inherent constraint of NHID-based research, but it does not materially affect the population-level trends that were the focus of this study.”

Comment 2: The authors mentioned “suggesting that rising cataract surgeries likely contributed to the decline in LPI use.” However, since the analysis could not distinguish between the two eyes of a single patient and was based only on incidence data, it seems difficult to draw this causal conclusion.

Response: Thank you for this valuable comment. We agree that the original phrase (“likely contributed”) may have implied a causal relationship, which cannot be supported by our incidence-based, person-level analysis that lacks laterality information. Our intent was to describe a temporal association, not causality.

Accordingly, we have revised the sentence in the Discussion to reflect associative—rather than causal—language, in line with the descriptive nature of our study.

• Discussion (Line 229):

Original: “The rates per 100,000 population mirrored this pattern, suggesting that rising cataract surgeries likely contributed to the decline in LPI use.”

Revised: “The rates per 100,000 individuals in this age group mirrored this pattern, indicating an inverse temporal association between cataract surgeries and LPI procedures over the study period.”

Comment 3: The statement “The annual number of patients who underwent cataract surgery or LPI for PACG was analyzed, along with the rate of these procedures per 100,000 population” (lines 112-113) — does this refer to the rate among the entire Korean population, or only among those aged 65 years and older?

Response: Thank you for raising this important point. The rates per 100,000 population in our study were calculated using the annual population of adults aged 65 years and older, not the entire Korean population. Because our cohort was restricted to individuals aged 65 and above, we used the corresponding age-specific population from Statistics Korea to ensure appropriate standardization.

We revised the Methods and Results sections accordingly.

• Methods (Line 113):

Original: “The annual number of patients who underwent cataract surgery o

Attachment Submitted filename: Response to Reviewers.docx

# Peer review: Decision Letter 1

**DOI:** 10.1371/journal.pone.0343427.r003

**Authors:** Daniel Duck-Jin Hwang

5 Feb 2026

Declining laser peripheral iridotomy for angle closure alongside rising cataract surgeries: A nationwide cohort study in South Korea

PONE-D-25-49693R1

Dear Dr. Song,

We’re pleased to inform you that your manuscript has been judged scientifically suitable for publication and will be formally accepted for publication once it meets all outstanding technical requirements.

Within one week, you’ll receive an e-mail detailing the required amendments. When these have been addressed, you’ll receive a formal acceptance letter and your manuscript will be scheduled for publication.

An invoice will be generated when your article is formally accepted. Please note, if your institution has a publishing partnership with PLOS and your article meets the relevant criteria, all or part of your publication costs will be covered. Please make sure your user information is up-to-date by logging into Editorial Manager at Editorial Manager®  and clicking the ‘Update My Information' link at the top of the page. For questions related to billing, please contact billing support .

If your institution or institutions have a press office, please notify them about your upcoming paper to help maximize its impact. If they’ll be preparing press materials, please inform our press team as soon as possible -- no later than 48 hours after receiving the formal acceptance. Your manuscript will remain under strict press embargo until 2 pm Eastern Time on the date of publication. For more information, please contact onepress@plos.org.

Kind regards,

Daniel Duck-Jin Hwang

Academic Editor

PLOS One

Additional Editor Comments:

The authors have revised and supplemented their manuscript appropriately. Thank you for the diligent revisions made to the manuscript in line with our comments. I truly appreciate your hard work and dedication to improving the manuscript.

Reviewers' comments:

Reviewer's Responses to Questions

**Comments to the Author**

Reviewer #1: All comments have been addressed

Reviewer #2: All comments have been addressed

**********

2. Is the manuscript technically sound, and do the data support the conclusions?

Reviewer #1: Yes

Reviewer #2: Yes

**********

3. Has the statistical analysis been performed appropriately and rigorously?

Reviewer #1: Yes

Reviewer #2: Yes

**********

4. Have the authors made all data underlying the findings in their manuscript fully available?

The PLOS Data policy

Reviewer #1: Yes

Reviewer #2: Yes

**********

5. Is the manuscript presented in an intelligible fashion and written in standard English?

Reviewer #1: Yes

Reviewer #2: Yes

**********

Reviewer #1: Authors addressed replies well and revised their manuscript accordingly based on the reviewer's raised concerns.

Reviewer #2: The revised manuscript has adequately addressed all reviewer's questions.

No further comments for this manuscript.

**********

what does this mean? ). If published, this will include your full peer review and any attached files.

If you choose “no”, your identity will remain anonymous but your review may still be made public.

**Do you want your identity to be public for this peer review?** For information about this choice, including consent withdrawal, please see our Privacy Policy

Reviewer #1: No

Reviewer #2: No

**********

# Editor report: Acceptance letter

**DOI:** 10.1371/journal.pone.0343427.r004

**Authors:** Daniel Duck-Jin Hwang

PONE-D-25-49693R1

PLOS One

Dear Dr. Song,

I'm pleased to inform you that your manuscript has been deemed suitable for publication in PLOS One. Congratulations! Your manuscript is now being handed over to our production team.

At this stage, our production department will prepare your paper for publication. This includes ensuring the following:

* All references, tables, and figures are properly cited

* All relevant supporting information is included in the manuscript submission,

* There are no issues that prevent the paper from being properly typeset

You will receive further instructions from the production team, including instructions on how to review your proof when it is ready. Please keep in mind that we are working through a large volume of accepted articles, so please give us a few days to review your paper and let you know the next and final steps.

Lastly, if your institution or institutions have a press office, please let them know about your upcoming paper now to help maximize its impact. If they'll be preparing press materials, please inform our press team within the next 48 hours. Your manuscript will remain under strict press embargo until 2 pm Eastern Time on the date of publication. For more information, please contact onepress@plos.org.

You will receive an invoice from PLOS for your publication fee after your manuscript has reached the completed accept phase. If you receive an email requesting payment before acceptance or for any other service, this may be a phishing scheme. Learn how to identify phishing emails and protect your accounts at https://explore.plos.org/phishing.

If we can help with anything else, please email us at customercare@plos.org.

Thank you for submitting your work to PLOS ONE and supporting open access.

Kind regards,

PLOS ONE Editorial Office Staff

on behalf of

Professor Daniel Duck-Jin Hwang

Academic Editor

PLOS One
